# De Novo Immune Induction After COVID‐19 Vaccination Under B‐Cell Depletion Is Characterized by Robust T‐Cellular Immunity in Patients With Inflammatory Central Nervous System Disease

**DOI:** 10.1002/brb3.70849

**Published:** 2025-09-02

**Authors:** Mathias Fousse, Verena Klemis, Saskia Bronder, Rebecca Urschel, Franziska Hielscher, Klaus Faßbender, Urban Sester, Martina Sester, Tina Schmidt

**Affiliations:** ^1^ Department of Neurology Saarland University Homburg Germany; ^2^ Department of Transplant and Infection Immunology Saarland University Homburg Germany; ^3^ SHG Kliniken Völklingen Germany

**Keywords:** B‐cell depletion, multiple sclerosis, SARS‐CoV‐2, T cells, vaccination

## Abstract

**Background:**

Immune induction under B‐cell depletion is complex and far from being fully understood.

**Methods:**

We investigated clinical and immunological responses after dual homologous mRNA vaccination with BNT162b2 and after booster vaccination or infection in 14 B‐cell depleted patients with inflammatory central nervous system disease in comparison to 28 healthy controls. Spike‐specific IgG were determined using ELISA and neutralizing activity by surrogate assay. Reactive T cells were flow‐cytometrically analyzed after spike‐specific and polyclonal stimulation. Reactogenicity was self‐reported using a questionnaire.

**Results:**

Vaccination was well tolerated, with slightly more systemic events reported by patients. Spike‐specific antibodies were induced in all controls, but only 43% of patients with significantly lower IgG levels and reduced neutralizing capacity (*p* < 0.0001). In contrast, spike‐reactive T cells were induced in both groups with similar CD4 and higher CD8 T‐cell levels in patients. Functional characterization of spike‐reactive T cells revealed equally high CTLA‐4 expression in both groups, but higher proportions of polyfunctional, triple‐cytokine expressing CD4 and CD8 T cells in patients especially after the third immunization. Three patients experienced mild breakthrough infections after second vaccination.

**Conclusions:**

Despite limited ability of B‐cell depleted patients to mount a humoral immune response after multiple doses of SARS‐CoV‐2 mRNA vaccination, the vaccine‐induced T‐cell response is robust, which may have implications for protection against severe disease.

## Graphical abstract

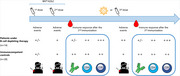

## 1 Introduction

The severe acute respiratory syndrome coronavirus 2 (SARS‐CoV‐2) emerged in December 2019 as a new member of the coronavirus family causing the COVID‐19 pandemic. The newly developed vaccines against SARS‐CoV‐2 were effective in immunocompetent individuals, with mRNA‐based vaccines encoding the SARS‐CoV‐2 spike protein among the most commonly applied vaccines to mitigate the risk of infection and severe disease progression (Polack et al. 2020). After translation of the mRNA in host cells, a spike‐specific humoral and cellular immune response is induced (Polack et al. 2020). Both the mRNA‐1273 (Moderna) and BNT162b2 (BioNTech/Pfizer) were shown to induce high titers of spike protein‐binding antibodies with neutralizing activity and specific CD4 and CD8 T cells in healthy immunocompetent individuals (Sahin et al., 2021).

The individual vaccine‐induced immune response may be impaired due to underlying diseases and immunosuppressive medication (Disanto et al. 2023). Many autoimmune neurological diseases such as multiple sclerosis (MS) and neuromyelitis optica spectrum disorder (NMOSD) are based on B‐cell or antibody‐mediated pathophysiology (Lee et al. 2021; Sabatino et al. 2019). Therefore, monoclonal antibodies directed against CD20, a surface‐antigen on precursor, mature and memory B cells including rituximab and ocrelizumab, are currently used for the treatment of these autoimmune diseases. Intravenous application causes a rapid and sustained long‐lasting depletion of circulating B cells (Sabatino et al. 2019).

With continued anti‐CD20 therapy, persistent B‐cell depletion (BCD) decreases IgG fractions, thereby increasing the risk of infections (Barmettler et al. 2018). In addition, immunogenicity and vaccination efficacy may be decreased. Moreover, patients with MS and NMOSD generally feel discomfort in getting vaccinated due to a potential risk of disease relapses (Stastna et al. 2022). Therefore, assessing immunogenicity and vaccine efficacy in these patients is crucial to balance the potential risk of relapses after vaccination against its benefit of reducing the risk of severe disease due to infections, or infection‐associated relapses (Stastna et al. 2022). Moreover, primary vaccination of B‐cell depleted patients against SARS‐CoV‐2 allows the possibility to study a de novo induction of antigen‐specific cellular immunity in the absence of B cells and antibodies. We therefore performed a detailed quantitative, phenotypical, and functional analysis of vaccine‐induced spike‐specific cellular immune responses in neurological patients undergoing BCD therapy and immunocompetent individuals after primary homologous mRNA‐based vaccination and following booster immunization.

## 2 Patients and Methods

### 2.1 Study Design and Participants

Fourteen patients receiving BCD therapy due to different etiologies of neurologic autoimmune diseases were prospectively enrolled at the Department of Neurology of the Saarland University Medical Center. Twenty‐eight immunocompetent healthy individuals matched for age, sex, vaccine regimen of primary vaccination, and time between vaccinations and analyses served as controls. All study participants initially received two doses of the mRNA‐based vaccine BNT162b2 (BioNTech/Pfizer) within a 3‐ to 6‐week interval according to German recommendations. A third vaccination was performed with either BNT162b2 or mRNA‐1273 (Moderna). Three patients had a SARS‐CoV‐2‐infection after the second vaccination and did not receive a third dose, but a sample was tested after infection. Two patients were lost to follow‐up after the third vaccination. Blood samples were analyzed 13–27 days after the second vaccination and 10–64 days after the third vaccination or infection. Reactogenicity within 7 days after vaccination was self‐reported using a standardized questionnaire.

The study was approved by the local ethics committee (Ethikkommission der Ärztekammer des Saarlandes, No. 76/20), and all study participants provided written informed consent. Ongoing viral or bacterial infection and a known history of SARS‐CoV‐2 infection or vaccination were defined as exclusion criteria. The study was conducted from March 2021 to April 2022.

### 2.2 Quantification of Leukocyte Subpopulations

Numbers of leukocytes and subpopulations were extracted from differential blood counts. The proportion of lymphocyte subpopulations was analyzed by flow cytometry after surface staining, either as part of clinical routine analyses for patients or using anti‐CD3 (clone SK7, RRID:AB_2783791), anti‐CD4 (SK3, RRID:AB_1645732), anti‐CD8 (RPA‐T8, RRID:AB_1937325), and anti‐CD19 (HIB19, RRID:AB_395812) for controls.

### 2.3 Quantification and Characterization of SARS‐CoV‐2 Spike‐Specific T Cells

Quantification as well as phenotypical and functional characterization of SARS‐CoV‐2 spike‐specific CD4 and CD8 T cells was performed as previously described (Schmidt et al. 2021a, 2021b). In brief, heparinized whole blood samples were stimulated with overlapping peptides spanning the SARS‐CoV‐2 spike protein (2 µg/mL/peptide; JPT, Berlin, Germany). Peptide diluent (0.64% DMSO) served as negative control, and 2.5 µg/mL *Staphylococcus aureus* enterotoxin B (SEB; Sigma) served as a polyclonal stimulus and positive control. Cells were stimulated for 6 h in the presence of co‐stimulatory antibodies against CD49d and CD28 (1 µg/ml each), followed by fixation and immunostaining with anti‐CD4 (clone SK3, RRID:AB_1645732), anti‐CD8 (clone SK1, RRID:AB_2868802), anti‐IFNγ (clone 4S.B3, RRID:AB_395473), anti‐CD69 (clone L78, RRID:AB_1937286), anti‐CTLA‐4 (clone BNI3, RRID:AB_398615), anti‐IL‐2 (clone MQ1‐17H12, RRID:AB_397231), and anti‐TNF (clone MAb11, RRID:AB_10646031).

SARS‐CoV‐2‐reactive CD4 or CD8 T cells were detected using flow cytometry (on a FACS Canto II cytometer and FACSDiva software 6.1.3) as CD69‐IFNγ‐double positive T cells as previously described (Schmidt et al. 2021a). Multifunctional cells were identified by co‐expression of IFNγ, IL‐2, and TNF, and cytokine expression profiles were determined by dividing CD69+ cytokine‐expressing cells into seven subpopulations of cells expressing one, two, or three cytokines simultaneously (Schmidt et al. 2021a). Percentages of reactive SARS‐CoV‐2‐specific T cells were defined after subtraction of respective percentages of reactive cells after negative control stimulation. A detection limit of 0.03% reactive cells was used as established before (Schmidt et al. 2021a).

### 2.4 Determination of SARS‐CoV‐2 Spike‐Specific Antibodies and Neutralizing Activity

IgG antibodies toward the receptor‐binding domain (RBD) of SARS‐CoV‐2 spike protein were quantified using an enzyme‐linked immunosorbent assay (ELISA) according to the manufacturer's instructions (SARS‐CoV‐2‐QuantiVac, Euroimmun, Lübeck, Germany). Neutralizing antibodies were analyzed in individuals with SARS‐CoV‐2‐positive IgG levels using a neutralization assay according to the manufacturer's instructions (SARS‐CoV‐2‐NeutraLISA, Euroimmun). Quantification of neutralizing activity was based on antibody‐mediated inhibition of soluble ACE2 binding to the plate‐bound spike‐RBD, and neutralizing capacity was expressed as percentage of inhibition (IH, calculated by 1 minus the ratio of the extinction of the patient sample and the blank value).

### 2.5 Statistical Analysis

The Mann–Whitney test was performed to compare unpaired non‐parametric data between groups (time since vaccination, lymphocyte subpopulations, T‐cell and antibody levels, CTLA‐4 expression levels). Comparisons of paired data of antibody and T‐cell levels after the second and third immunization were performed using Wilcoxon signed‐rank test. Data with normal distribution were analyzed using unpaired *t*‐test (age, cytokine‐expression profiles). Categorical analyses of sex and reactogenicity were performed using Fisher's exact test. Correction for multiple testing was carried out according to Benjamini and Yekutieli with maximum 5% false discovery rate. A *p* value < 0.05 was considered statistically significant. Statistical analysis was carried out using the GraphPad Prism 10.0.3 software (GraphPad, San Diego, CA, USA) using two‐tailed tests.

## 3 Results

### 3.1 Study Population

Fourteen patients on B‐cell depleting therapy and 28 matched healthy immunocompetent controls were recruited for analyses of spike‐specific immunity after the second and third immunization (Table 1). Among the patients, 10 had MS, three had an NMOSD, and one had an antineutrophil cytoplasmic antibodies (ANCA)‐associated vasculitis. All 10 MS patients received 600 mg of ocrelizumab semiannually (after loading dose of 300 mg on day 1 and day 15), and the four other patients with NMOSD and ANCA‐associated vasculitis received rituximab only (1000 mg semiannually after a loading dose of 1000 mg on day 1 and day 15). One NMOSD patient was pregnant during the first two vaccinations and was one of the two patients who were lost to follow‐up after the third vaccination. Disability among patients was scored with a median of 3.5 (IQR 3.5) on the expanded disability status scale (EDSS). In all patients, BCD had been established before SARS‐CoV‐2 vaccination initiation. The median interval between the last BCD infusion and the first immunizations was 107.5 (IQR 48.8) days (first vaccination), 147.5 (IQR 43.0) days (second vaccination with no patient receiving a BCD infusion between the first and second vaccination), and 124.0 (IQR 68.0) days (third vaccination/infection), respectively. All patients remained under BCD therapy throughout follow‐up. Further immune‐modifying therapies are shown in Table 1. Three patients (all under treatment with ocrelizumab) acquired infection with SARS‐CoV‐2 with only mild symptoms 111, 141, and 168 days after the second vaccination. The patient infected after 111 days was first infected with the delta variant, followed by re‐infection with the omicron variant 68 days later. Genotyping of the viral variant was not performed in the two other patients.

**TABLE 1:** Demographic and clinical characteristics of the study population.

	Patients	Controls	
	*n* = 14	*n* = 28	*p* value
Years of age, mean ± SD	44.7 ± 13.6	51.3 ± 12.8	0.132
Female sex, *n* (%)	7 (50.0)	14 (50.0)	> 0.999
Analysis time after second vaccination, median days (IQR)	20 (6)	17 (7)	0.197
Analysis time after third antigen contact, median days (IQR)^a^	31.0 (23.8)	30.5 (35.8)	0.959
Underlying disease, *n* (%)			
RMS/SPMS	4 (28.6)	n.a.	
RRMS	6 (42.9)	n.a.	
NMOSD^b^	3 (21.4)	n.a.	
ANCA‐associated vasculitis	1 (7.1)	n.a.	
Years of disease duration, median (IQR)	5.0 (20.8)	n.a.	
Disability, EDSS (0–10), median (IQR)	3.5 (3.5)	n.a.	
B‐cell depleting medication, *n* (%)			
Ocrelizumab (RMS/SPMS, RRMS)	10 (71.4)	n.a.	
Rituximab (NMOSD, ANCA‐associated vasculitis)	4 (28.6)	n.a.	
Number of infusion cycles, median (IQR)	3.5 (5.3)	n.a.	
≤5, *n* (%)	10 (71.4)	n.a.	
> 5, *n* (%)	4 (28.6)	n.a.	
Concomitant prednisolone therapy, *n* (%)	2^c^ (14.3)	n.a.	
Previous immunotherapies,^d^ median number (IQR)	1.5 (1.8)	n.a.	
Differential blood cell counts (cells/µL), median (IQR)	*n* = 14	*n* = 26	
Leukocytes	6950 (2250)	6000 (2150)	0.136
Granulocytes^e^	4579 (1437)	3513 (1447)	0.030
Monocytes^e^	552 (313)	509 (212)	0.294
Lymphocytes	1572 (1219)	2063 (719)	0.138
CD3 T cells^f^	1371 (961)	1180 (854)	0.780
CD4 T cells^f^	755 (606)	871 (519)	0.451
CD8 T cells^f^	543 (437)	327 (154)	0.018
B cells^f^, ^g^	0 (0)	173 (179)	< 0.0001

*Note*: If not indicated otherwise, data refer to the time point after the second vaccination.

Abbreviations: ANCA, antineutrophil cytoplasmic antibodies; EDSS, expanded disability status scale (from 0 to 10); NMOSD, neuromyelitis optica spectrum disorder; RMS, relapsing multiple sclerosis; RRMS, relapsing‐remitting multiple sclerosis; SPMS, secondary progressive multiple sclerosis.

^a^ Samples of 12 patients were available after third antigen contact (nine due to vaccination, three due to infection).

^b^ NMOSD patients: one detection of aquaporin‐4 antibodies, one detection of anti‐MOG antibodies, and one antibody negative.

^c^ 20 mg and 50 mg daily, respectively.

^d^ Immunotherapeutics for long‐term treatment without corticosteroids, including cyclophosphamide (*n* = 2) and mitoxantrone (*n* = 2).

^e^ Granulocyte and monocyte counts were calculated on 12 patients and 26 controls.

^f^ Counts of lymphocyte subpopulations were calculated on 14 patients and 15 controls.

^g^ Circulating B cells were defined as CD19^+^CD3^−^ lymphocytes.

Comparison of leukocyte subpopulations between patients and controls confirmed depletion of B cells in the patient group with very low B‐cell counts in three patients only (one before the first vaccination with 22 cells/µL, one after the second vaccination with 22 cells/µL, and one after the third vaccination with 10 cells/µL). Moreover, patients had significantly higher levels of granulocytes (*p* = 0.030) and CD8 T cells (*p* = 0.018, Table 1).

### 3.2 Slightly More Pronounced Reactogenicity After Vaccination in B‐Cell Depleted Patients

**FIGURE 1:**
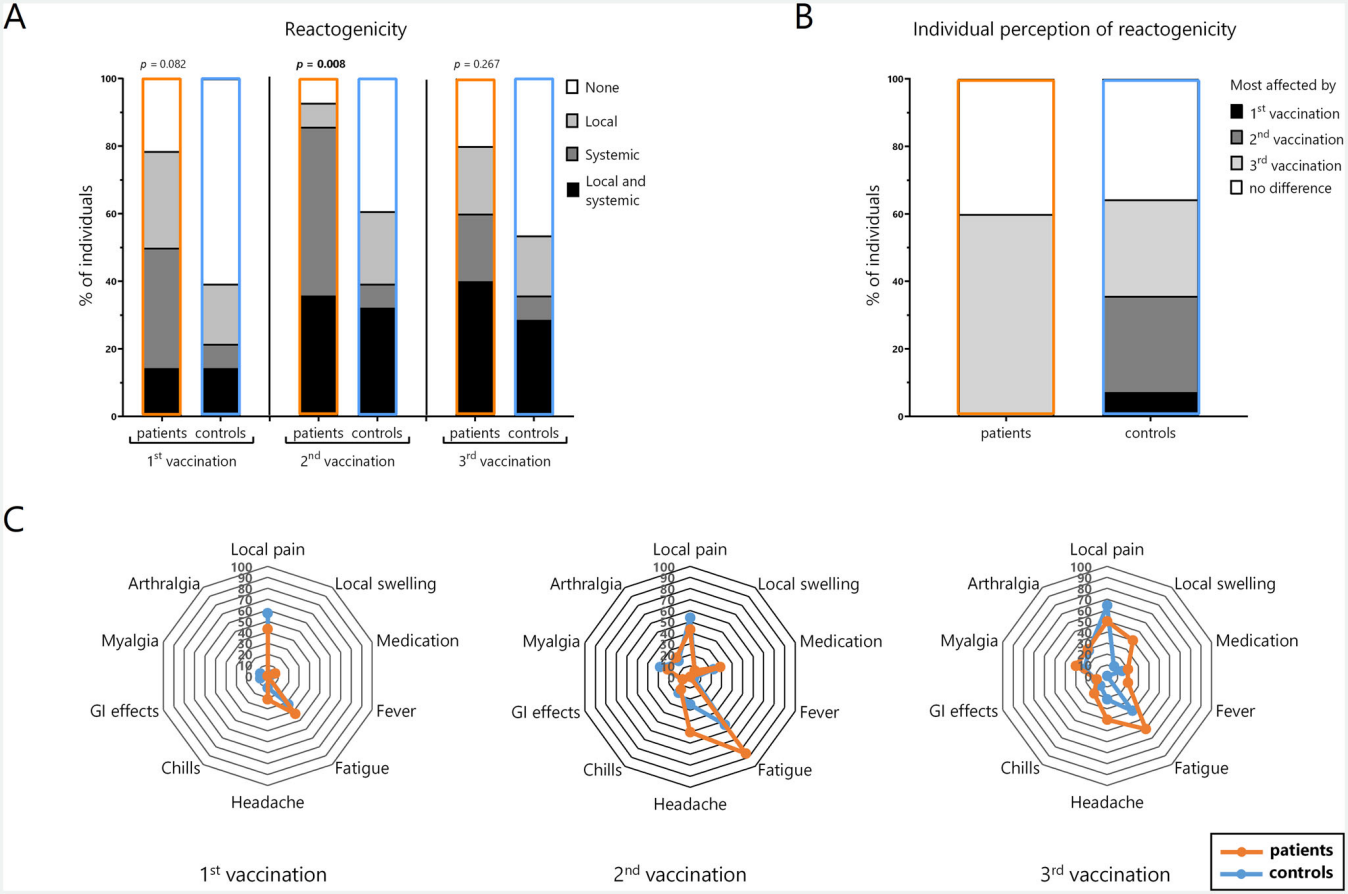
Reactogenicity after vaccination of B‐cell depleted patients and healthy controls. Results of the self‐reporting via standardized questionnaires addressing (A) differentiation between no, local, systemic, and combined symptoms; (B) individual perception of which vaccination was most affecting; and (C) percentage of patients (orange) and controls (blue) with specific symptoms. Data were available of all 14 patients and 28 controls after the first and the second vaccination and of 10 patients and 28 controls after the third vaccination. *p* values in (A) were determined by comparing the number of patients and controls with none/local and systemic symptoms (alone or in combination with local events) using Fisher's exact test; *p* values < 0.05 were considered significant.

Reactogenicity during the first week after each vaccination was self‐reported using a standardized questionnaire. In both groups, the proportion of individuals without symptoms was the highest after the first vaccination, whereas the highest overall reactogenicity was reported after the second vaccination (Figure 1A). In general, systemic events were significantly more frequent in patients after the second vaccination (*p* = 0.008). Of note, 60% of patients felt most affected by the third vaccination, whereas the remaining 40% perceived no differences between the three vaccinations (Figure 1B). In contrast, the distribution of symptoms among controls was more balanced with only 29% of individuals reporting as having been most affected by the third vaccination.

Vaccinations were overall well tolerated in both groups with pain at the injection site, fatigue, and headache being the most prominent symptoms (Figure 1C, Figure S1). Although patients showed a trend toward a higher proportion with fatigue (*p* = 0.049) and with local swelling (*p* = 0.063) after the second and third vaccination, respectively, correction for multiple testing revealed no significant differences between the groups (Figure S1).

No patient suffered from a worsening of the neurological disease or disability (EDSS) after vaccination. The three patients who had a SARS‐CoV‐2 infection after their second vaccination had a clinically mild course with no need for hospitalization.

Taken together, although symptoms were generally mild, reactogenicity was slightly more frequent among B‐cell depleted patients, who felt most affected by the third vaccination.

### 3.3 Higher Percentages of SARS‐CoV‐2 Spike‐Specific CD8 T Cells in B‐Cell Depleted Patients Compared to Immunocompetent Controls

**FIGURE 2:**
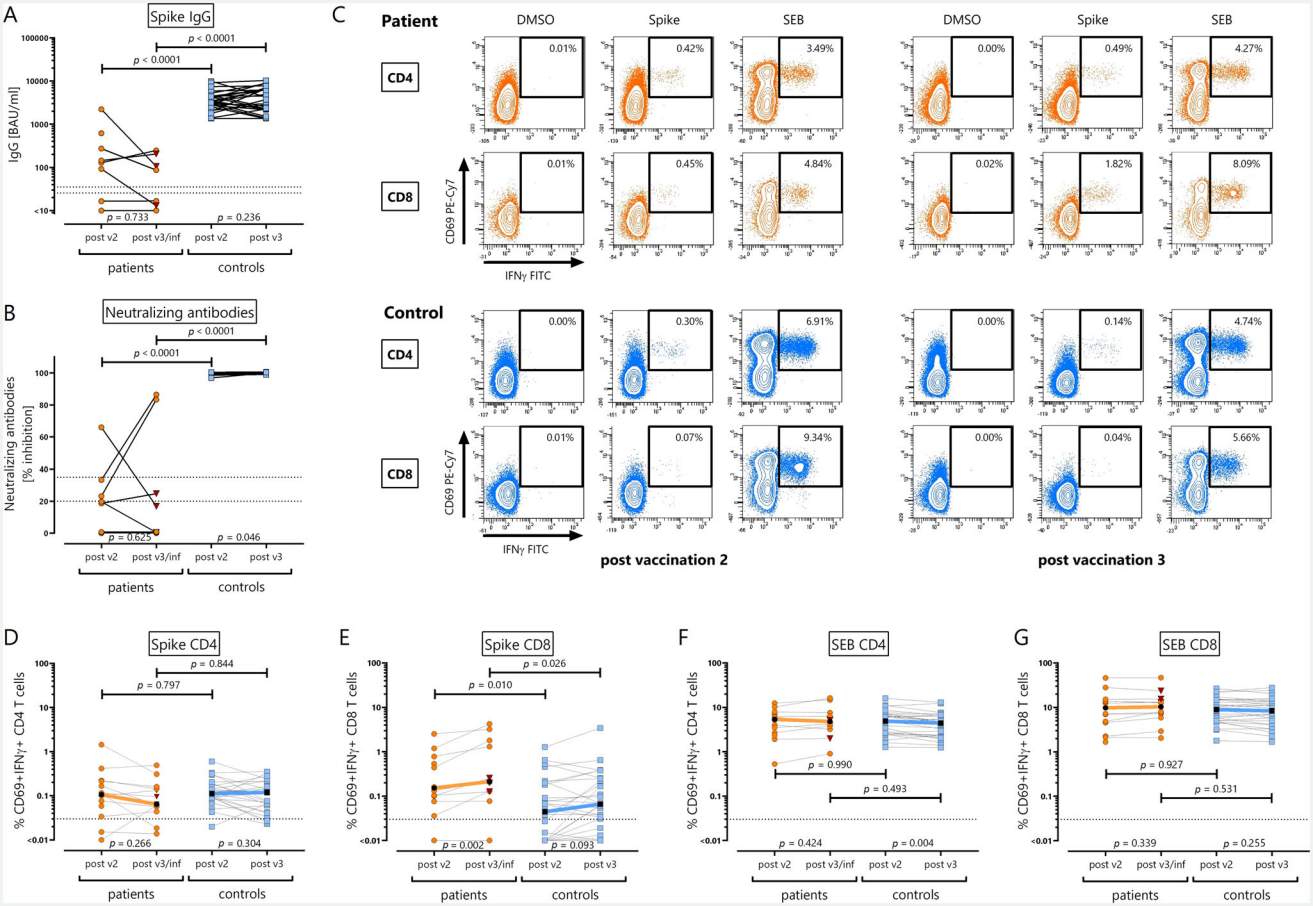
Antibody and T‐cell responses after the second and third immunization. (A) Levels of SARS‐CoV‐2 IgG were determined by ELISA, and (B) neutralizing antibody activity was expressed as % inhibition (IH) in the surrogate assay. Paired analyses after the second vaccination (post v2) and after the third vaccination (post v3) or infection (inf, red triangles) are shown. Dotted lines denote respective thresholds (positive ≥35.2 binding antibody units [BAU]/mL, intermediate < 35.2 and ≥25.6, and negative < 25.6 BAU/mL; neutralizing activity: positive result at IH ≥35, intermediate at IH < 35 and ≥20, and negative at IH<20). (C) Representative contour plots depicting flow cytometric analysis of reactive CD4 and CD8 T cells after stimulation with diluent (DMSO, negative control), overlapping peptides derived from SARS‐CoV‐2 spike and *Staphylococcus aureus* enterotoxin B (SEB, positive control). Shown are the results of a 25‐year‐old female B‐cell depleted patient (orange), and a 24‐year‐old female healthy control (blue). Numbers indicate percentages of reactive CD4 or CD8 T cells, defined by their co‐expression of CD69 and interferon gamma (IFNγ). Percentages of spike‐specific (CD69+IFNγ+) (D) CD4 and (E) CD8 T cells after subtraction of reactive cells after negative control stimulation are depicted after the second and third immunization event for patients (*n* = 14 and *n* = 12, respectively; orange) and controls (*n* = 28; blue) with connecting lines for each individual. Reactive CD4 and CD8 T cells after polyclonal stimulation with SEB are shown in (F) and (G), respectively. Results after infection are represented by red triangles. Bold lines are connecting the respective median values, and dotted lines indicate the detection limit of 0.03% for spike‐specific T cells as determined previously (Schmidt et al. 2021a). Statistical analyses of differences between results after second and third vaccination/infection were performed using Wilcoxon signed‐rank test among individuals who had paired analyses at both time points. Comparisons between patients and controls at each time point were performed of all available data using Mann–Whitney test. *p* values < 0.05 were considered significant.

After two vaccinations, all controls were SARS‐CoV‐2‐specific IgG positive, whereas only 6/14 patients had detectable IgG. Correspondingly, spike‐specific IgG levels were significantly higher in controls (median 3383 [IQR 1774] BAU/mL) than in patients (median 11 [IQR 175] BAU/ml, *p* < 0.0001, Figure 2A). After the third vaccination, antibody levels did not further increase in controls (*p* = 0.236). Likewise, neither a third vaccination nor infection led to any further seroconversion among patients, and IgG levels in 1/6 patients with seroconversion even decreased below detection limit (Figure 2A).

As with IgG levels, neutralizing antibody activity was significantly higher in controls after both the second and third vaccination (*p* < 0.0001, Figure 2B). While almost 100% virus inhibition was detected in controls after the second vaccination, which slightly increased after the third vaccination (*p* = 0.046), only two patients reached neutralizing activity levels above 80% after three immunization events (Figure 2B).

Spike‐specific CD4 and CD8 T cells were quantified after stimulation with overlapping peptides spanning the SARS‐CoV‐2 spike protein based on induction of CD69 and IFNγ with representative contour plots of a patient and a control after the second and third vaccination shown in Figure 2C. DMSO and the polyclonal stimulus SEB served as negative and positive controls, respectively. Unlike IgG, spike‐specific CD4 and CD8 T cells were clearly detectable in the majority of patients after the second and/or third immunization (Figure 2D,E). Moreover, when comparing patients and controls, the percentage of spike‐specific CD4 T cells did not significantly differ neither after the second vaccination (*p* = 0.797) nor after the third immunization (*p* = 0.844, Figure 2D). Interestingly, median spike‐specific CD8 T‐cell levels after the second and third immunization were even higher in patients (0.152 [IQR 0.503] % and 0.212 [IQR 1.55] %, respectively) than in controls (0.045 [IQR 0.190] %, *p* = 0.010 and 0.066 [0.220] %, *p* = 0.026, respectively). Moreover, CD8 T‐cell levels of patients were significantly higher after the third than after the second immunization (*p* = 0.002), whereas no pronounced further increase was observed in controls (*p* = 0.093, Figure 2E). Of note, immune induction after infection seemed to be similar to that after vaccination (Figure 2A–E, red triangles). Finally, these observations were specific for spike‐reactive CD8 T cells, as CD4 and CD8 T‐cell reactivity after SEB stimulation was similar in patients and controls and did not show any vaccine‐induced changes (Figure 2F,G). Analysis of reactive cells by co‐expression of CD69 and IL‐2 or TNF revealed similar results than with IFNγ‐expressing cells, although differences were slightly less pronounced (Figure S2). Interestingly, levels of spike‐specific T cells were significantly higher in individuals reporting systemic adverse events both for CD4 (*p* = 0.001 after the second vaccination) and especially for CD8 T cells (*p* = 0.0008 after the second and *p* = 0.008 after the third vaccination, Figure S3).

In summary, although the majority of patients under B‐cell depleting therapy had limited antibody responses after vaccination, all patients showed a strong induction of SARS‐CoV‐2 spike‐specific T cells, which was similar or even more pronounced than in controls.

### 3.4 Distinct Differences in Functional Characteristics of the Spike‐Specific T Cells Between Patients and Controls

**FIGURE 3:**
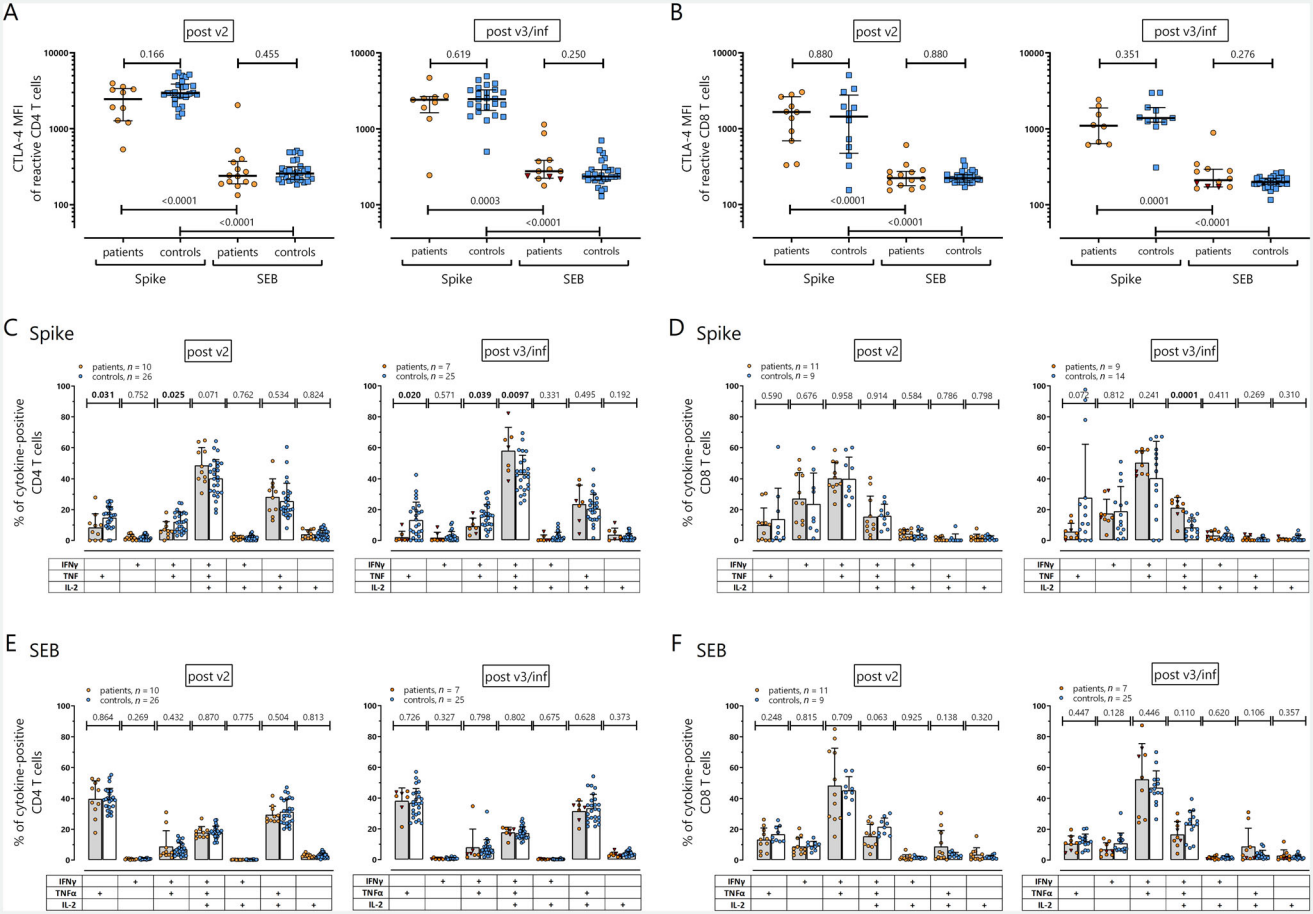
Cytokine and CTLA‐4 expression by spike‐specific and SEB‐reactive CD4 and CD8 T cells. CTLA‐4 median fluorescence intensities (MFI) of reactive (CD69+IFNγ+) CD4 (A) and CD8 T cells (B) after stimulation with SARS‐CoV‐2 spike peptides and *Staphylococcus aureus* enterotoxin B (SEB) were determined in all samples (with lack of relevant reactivity to DMSO) of B‐cell depleted patients (orange symbols) and healthy controls (blue symbols) with at least 20 CD69+IFNγ+ CD4 (*n* = 10 and 14 patients as well as 26 and 28 controls after the second vaccination; *n* = 9 and 12 patients as well as 24 and 28 controls after the third vaccination/infection) or CD8 T cells (*n* = 11 and 14 patients as well as 12 and 28 controls after the second vaccination; *n* = 8 and 12 patients as well as 11 and 28 controls after the third vaccination/infection). Bars represent median and interquartile range. Statistical analyses were performed using Mann–Whitney test, *p* < 0.05 was considered significant. Cytokine expression profiles of spike‐specific and SEB‐reactive (C and E) CD4 and (D and F) CD8 T cells showing single or combined expression of IFNγ, TNF and IL‐2 were determined of all samples with at least 30 cytokine‐expressing cells (numbers of included samples are indicated in the figure) and compared between patients (gray bars and orange symbols) and controls (white bars and blue symbols) using unpaired *t*‐test. Bars represent means and standard deviation; *p* < 0.05 were considered significant. Results after infection are represented by red triangular symbols.

To characterize spike‐specific T cells and qualitative differences between B‐cell depleted patients and controls in more detail, CTLA‐4 expression as well as cytokine profiles of spike‐specific T cells were directly compared after the second and third immunization (Figure 3A–D). Interestingly, in line with recent antigen encounter, spike‐specific CD4 and CD8 T cells of both patients and controls had significantly higher CTLA‐4 expression levels than SEB‐reactive cells with similarly high expression patterns after the second and third immunization (Figure 3A,B). However, CTLA‐4 levels of spike‐specific CD4 or CD8 T cells did not differ between patients and controls.

Cytokine profiling revealed a dominance in the percentage of triple cytokine positive cells among spike‐specific CD4 T cells, which was higher in patients than in controls, especially after the third immunization (Figure 3C). Patients showed concomitantly lower percentages of cells producing TNF alone or in combination with IFNγ, which was observed both after the second and third immunization. IL‐2 expression was generally low in CD8 T cells, and the cytokine profile was dominated by TNF and IFNγ (Figure 3D). Nevertheless, patients showed a clear proportion of triple‐cytokine positive CD8 T cells (mean ± sd 15.4 ± 13.3% after the second and 21.3 ± 6.7% after the third immunization), which was significantly higher compared to controls after the third immunization (*p* = 0.0001; Figure 3D). Again, the functional characteristics did not appear to be markedly influenced by the three infected patients (Figure 3A–D, red triangles). Moreover, those effects were specific for spike‐reactive T cells, as cytokine profiles after polyclonal stimulation did not show any differences between patients and controls (Figure 3E,F).

## 4 Discussion

To gain more insight in the mechanisms of de novo immune induction under B‐cell depleting conditions, we performed a detailed analysis of cellular immunity after primary homologous BNT162b2 vaccination and after booster by vaccination or infection of B‐cell depleted patients. Compared to matched healthy controls, humoral immunity was expectedly impaired. In contrast, vaccine‐induced SARS‐CoV‐2 spike‐specific CD4 and CD8 T cells were detectable in all patients with similar or even higher frequencies than in controls. Moreover, the percentage of multifunctional T cells with strong expression of CTLA‐4 as evidence for recent antigen encounter was higher in patients. In line with higher T‐cell responses, self‐reported systemic adverse events after each vaccination appeared to be slightly more frequent in patients than in controls.

In general, vaccination was well tolerated in all participants. Pain at the injection site, fatigue, and headache were the most common side effects, consistent with other studies of SARS‐CoV‐2 vaccine tolerability in MS patients (Stastna et al. 2022; Wieske et al. 2022). However, patients showed a trend toward a higher rate of systemic events than controls. In this and in a previous study including immunocompetent individuals and transplant recipients, we showed that vaccine‐induced T‐cell levels were significantly higher in individuals with systemic events (Schmidt et al. 2021b). Hence, given the stronger T‐cell response in B‐cell depleted patients, the trend toward a higher reactogenicity may be indicative of a more intense immunological challenge.

The rate of patients with detectable humoral immune response, including spike‐specific IgG and neutralizing antibody activity, in B‐cell depleted patients was significantly lower compared to controls. This is in line with previous reports, where the anti‐CD20 therapy was identified as the main cause of reduced antibody response (Etemadifar et al. 2022; Hammer et al. 2022), and induction of a humoral immune response was shown to be positively correlated with the time since last infusion (Brill et al. 2021). In our study, all six patients with detectable antibody induction after the second vaccination received their vaccination at least 4 months after the last B‐cell depleting infusion, whereas this interval was below 3 months in three of eight IgG‐negative patients. This underlines the assumption that the induction of humoral immunity may be positively influenced by adequate timing between therapy infusion and vaccination (Brill et al. 2021).

Despite impaired humoral immunity, the majority of both controls and patients showed robust induction of spike‐reactive T cells. Consistent with our findings, limited antibody induction but pronounced vaccine‐specific T‐cellular immunity after SARS‐CoV‐2 mRNA vaccination with stability on follow‐up was shown for several B‐cell depleted patient cohorts on various immunosuppressive regimens (Bajwa et al. 2022; Gadani et al. 2021; Iannetta et al. 2021; Kister et al. 2024; Oyaert et al. 2022; Tortorella et al. 2022). Interestingly, this pronounced T‐cell reactivity seems to be a peculiarity of B‐cell depleting therapy as these patients show increased levels of IFNγ‐producing cells after spike‐specific stimulation when compared to MS patients without or with other disease‐modifying therapies (Gadani et al. 2021). For CD8 T cells, we even detected significantly higher percentages of reactive cells after both the second and third immunization when compared to controls, hinting toward particular BCD‐associated differences in vaccine‐induced cellular immunity. Our data are in line with Apostolidis and colleagues who also found increased CD8 responses, robust CD4 T‐cell immunity and compromised circulating T‐follicular helper cells after second vaccination in B‐cell depleted MS patients, especially in those lacking anti‐RBD IgG (Apostolidis et al. 2021). Apart from quantitative differences after both the second and third vaccination, our study also included functional characterization of the vaccine‐induced T cells based on cytokine profiling and revealed significantly higher percentages of multifunctional IFNγ, TNF, and IL‐2 co‐expressing CD4 and CD8 T cells in patients than in controls especially after the third immunization. Thus, it is tempting to speculate that B‐cell depleted patients experience a stronger benefit from a booster vaccination than immunocompetent individuals. In line with this, the third vaccine dose was identified as a variable reducing the risk of infection in an Italian multicenter study including 1855 patients with MS (Sormani et al. 2022). Moreover, the incidence of infections requiring hospitalization had decreased from 12.8% in the pre‐vaccination era to 3.1% after vaccination (Sormani et al. 2022). Apart from protection from infection, which is known to be mainly mediated by humoral immunity, a strong cellular immunity is discussed as potentially mediating protection from severe disease (GeurtsvanKessel et al. 2022; Moss 2022; Tarke et al. 2022). Although cellular immunity was not concomitantly assessed in the Italian study, one may hypothesize that spike‐specific cellular immunity and/or a particular dominance of specific CD8 T cells may have mediated protection from severe disease in the relative absence of humoral immunity. This is supported by observations in patients with hematologic cancer including those receiving anti‐CD20 therapy, where a higher number of CD8 T cells was associated with improved survival in an early study in 100 non‐vaccinated cancer patients, who have been hospitalized for COVID‐19 (Bange et al. 2021).

So far, knowledge on the underlying mechanisms for the strong induction of cellular immunity under BCD is limited. The relative absence of spike‐specific antibodies may contribute to prolonged availability of antigen for T‐cell stimulation. In line with this hypothesis, spike‐specific CD4 and CD8 T cells showed increased expression of CTLA‐4 when compared to polyclonally activated cells. This can be interpreted as sign of recent antigen encounter by vaccination, similar to dynamic increases of CTLA‐4 expression on VZV‐specific CD4 T cells during active infections observed in both patients with herpes zoster and VZV‐mediated infections of the central nervous system (Schub et al. 2018, 2015). Finally, a potential mechanism underlying the stronger CD8 response in B‐cell depleted patients may be based on an increased CD40‐CD40L interaction between CD8 and CD4 T cells, which is shown to improve the activation and formation of memory CD8 T cells (Ahmed et al. 2012; Ara et al. 2018). Unfortunately, neither CD40 nor CD40L have been analyzed in our patients, but may be of interest for future studies.

Our study is limited by a small sample size. Nevertheless, we not only confirmed results on robust vaccine‐induced cellular immune responses in B‐cell depleted patients, but also revealed specific functional characteristics of spike‐specific CD4 and CD8 T cells in direct comparison to matched controls. Moreover, our patient group comprised B‐cell mediated inflammatory diseases with heterogeneous underlying pathophysiological mechanisms. Despite this heterogeneity, the commonality that these patients share is the presence of a proven or suspected B‐cell pathology, which serves as a rationale for the administration of a treatment regimen involving anti‐CD20 antibodies. From an immunological point of view, our intention was therefore to study de novo vaccine‐induced immune responses in patients at the stage of B‐cell depletion with a particular focus on T‐cellular immunity. Overall, our observations of a robust vaccine‐induced cellular immune response provide a rational basis for clinical observations of vaccine‐mediated protection from severe disease despite considerable lack of antibodies. Future studies should address characterization of vaccine‐induced T cells as a correlate of protection, which is of particular relevance to patients with humoral immunodeficiencies.

In conclusion, the present study in patients with neuroimmunological diseases under B‐cell depleting therapy showed that de novo immunization against SARS‐CoV‐2 results in the induction of a robust and sustained T‐cell response. Despite the relative lack of a humoral immune response that would confer protection from infection, our findings of a preserved cellular immune response may suggest a potential protective effect against severe COVID‐19 disease. In this regard, the flow‐cytometric approach that we used to characterize the de novo vaccine‐induced T cells may broaden our knowledge on further refining cellular correlates of protection, and may be developed as a diagnostic assay to characterize vaccine‐induced T‐cell immunity in a clinical setting.

## Author Contributions

**Mathias Fousse**: writing – review and editing, writing – original draft, conceptualization, project administration, supervision, resources, formal analysis, validation, data curation. **Verena Klemis**: writing – review and editing, investigation. **Saskia Bronder**: writing – review and editing, formal analysis, visualization. **Rebecca Urschel**: writing – review and editing, investigation. **Franziska Hielscher**: writing – review and editing, investigation. **Klaus Faßbender**: writing – review and editing, resources. **Urban Sester**: writing – review and editing, methodology, resources. **Martina Sester**: writing – review and editing, writing – original draft, conceptualization, methodology, supervision, funding acquisition, project administration, data curation. **Tina Schmidt**: writing – review and editing, investigation, writing – original draft, conceptualization, methodology, validation, formal analysis, supervision, visualization, funding acquisition, project administration, data curation.

## Ethics Statement

All procedures performed in this study involving human participants were in accordance with the ethical standards of the institutional research committee and with the 1964 Helsinki Declaration and its later amendments or comparable ethical standards. The study was approved by the local ethics committee (Ethikkommission der Ärztekammer des Saarlandes, No. 76/20).

## Conflicts of Interest

T.S. has received travel grant support from Biotest outside the submitted work. M.S. received grant support from Biotest, Takeda, and Astellas to the organization Saarland University outside the submitted work, and honoraria for lectures from Biotest, Novartis, Takeda, MSD, and for participation in advisory boards from Biotest, Moderna, MSD and Takeda outside the submitted work. All other authors declare no conflicts of interest.

## Peer Review

The peer review history for this article is available at https://publons.com/publon/10.1002/brb3.70849.

## Supporting information




**Supplementary Materials**: brb370849‐sup‐0001‐SuppMat.docx

## Data Availability

All figures have associated raw data. The data that support the findings of this study are available from the corresponding author upon reasonable request.

## References

[brb370849-bib-0001] Ahmed, K. A. , L. Wang , M. A. Munegowda , et al. 2012. “Direct in Vivo Evidence of CD4^+^ T Cell Requirement for CTL Response and Memory via pMHC‐I Targeting and CD40L Signaling.” Journal of Leukocyte Biology 92, no. 2: 289–300. 10.1189/jlb.1211631. 22544940

[brb370849-bib-0002] Apostolidis, S. A. , M. Kakara , M. M. Painter , et al. 2021. “Cellular and Humoral Immune Responses Following SARS‐CoV‐2 mRNA Vaccination in Patients With Multiple Sclerosis on Anti‐CD20 Therapy.” Nature Medicine 27, no. 11: 1990–2001. 10.1038/s41591-021-01507-2. PMC8604727 34522051

[brb370849-bib-0003] Ara, A. , K. A. Ahmed , and J. Xiang . 2018. “Multiple Effects of CD40‐CD40L Axis in Immunity Against Infection and Cancer.” ImmunoTargets and Therapy 7: 55–61. 10.2147/ITT.S163614. 29988701 PMC6029590

[brb370849-bib-0004] Bajwa, H. M. , F. Novak , A. C. Nilsson , et al. 2022. “Persistently Reduced Humoral and Sustained Cellular Immune Response From First to Third SARS‐CoV‐2 mRNA Vaccination in Anti‐CD20‐Treated Multiple Sclerosis Patients.” Multiple Sclerosis and Related Disorders 60: 103729. 10.1016/j.msard.2022.103729. 35334278 PMC8898195

[brb370849-bib-0005] Bange, E. M. , N. A. Han , P. Wileyto , et al. 2021. “CD8^+^ T Cells Contribute to Survival in Patients With COVID‐19 and Hematologic Cancer.” Nature Medicine 27, no. 7: 1280–1289. 10.1038/s41591-021-01386-7. PMC8291091 34017137

[brb370849-bib-0006] Barmettler, S. , M. S. Ong , J. R. Farmer , H. Choi , and J. Walter . 2018. “Association of Immunoglobulin Levels, Infectious Risk, and Mortality with Rituximab and Hypogammaglobulinemia.” JAMA Network Open 1, no. 7: e184169. 10.1001/jamanetworkopen.2018.4169. 30646343 PMC6324375

[brb370849-bib-0007] Brill, L. , A. Rechtman , O. Zveik , et al. 2021. “Humoral and T‐Cell Response to SARS‐CoV‐2 Vaccination in Patients with Multiple Sclerosis Treated with Ocrelizumab.” JAMA Neurology 78, no. 12: 1510–1514. 10.1001/jamaneurol.2021.3599. 34554197 PMC8461553

[brb370849-bib-0008] Disanto, G. , A. Galante , M. Cantu , et al. 2023. “Longitudinal Postvaccine SARS‐CoV‐2 Immunoglobulin G Titers, Memory B‐Cell Responses, and Risk of COVID‐19 in Multiple Sclerosis Over 1 Year.” Neurology Neuroimmunology & Neuroinflammation 10, no. 1: e200043. 10.1212/NXI.0000000000200043. PMC9747147 36396447

[brb370849-bib-0009] Etemadifar, M. , H. Nouri , M. Pitzalis , et al. 2022. “Multiple Sclerosis Disease‐Modifying Therapies and COVID‐19 Vaccines: A Practical Review and Meta‐Analysis.” Journal of Neurology, Neurosurgery, and Psychiatry 93, no. 9: 986–994. 10.1136/jnnp-2022-329123. 35688629

[brb370849-bib-0010] Gadani, S. P. , M. Reyes‐Mantilla , L. Jank , et al. 2021. “Discordant Humoral and T Cell Immune Responses to SARS‐CoV‐2 Vaccination in People With Multiple Sclerosis on Anti‐CD20 Therapy.” EBioMedicine 73: 103636. 10.1016/j.ebiom.2021.103636. 34666226 PMC8520057

[brb370849-bib-0011] GeurtsvanKessel, C. H. , D. Geers , K. S. Schmitz , et al. 2022. “Divergent SARS‐CoV‐2 Omicron‐Reactive T and B Cell Responses in COVID‐19 Vaccine Recipients.” Science Immunology 7, no. 69: eabo2202. 10.1126/sciimmunol.abo2202. 35113647 PMC8939771

[brb370849-bib-0012] Hammer, H. , R. Hoepner , C. Friedli , et al. 2022. “Comparison of mRNA Vaccinations With BNT162b2 or mRNA‐1273 in Anti‐CD20‐Treated Multiple Sclerosis Patients.” Vaccines 10, no. 6: 922. 10.3390/vaccines10060922. 35746529 PMC9229998

[brb370849-bib-0013] Iannetta, M. , D. Landi , G. Cola , et al. 2021. “B‐ and T‐Cell Responses after SARS‐CoV‐2 Vaccination in Patients With Multiple Sclerosis Receiving Disease Modifying Therapies: Immunological Patterns and Clinical Implications.” Frontiers in Immunology 12: 796482. 10.3389/fimmu.2021.796482. 35111162 PMC8801814

[brb370849-bib-0014] Kister, I. , R. Curtin , A. L. Piquet , et al. 2024. “Longitudinal Study of Immunity to SARS‐CoV2 in ocrelizumab‐Treated MS Patients up to 2 Years After COVID‐19 Vaccination.” Annals of Clinical and Translational Neurology 11, no. 7: 1750–1764. 10.1002/acn3.52081. 38713096 PMC11251481

[brb370849-bib-0015] Lee, D. S. W. , O. L. Rojas , and J. L. Gommerman . 2021. “B Cell Depletion Therapies in Autoimmune Disease: Advances and Mechanistic Insights.” Nature Reviews Drug Discovery 20, no. 3: 179–199. 10.1038/s41573-020-00092-2. 33324003 PMC7737718

[brb370849-bib-0016] Moss, P. 2022. “The T Cell Immune Response Against SARS‐CoV‐2.” Nature Immunology 23, no. 2: 186–193. 10.1038/s41590-021-01122-w. 35105982

[brb370849-bib-0017] Oyaert, M. , M. A. de Scheerder , S. van Herrewege , et al. 2022. “Evaluation of Humoral and Cellular Responses in SARS‐CoV‐2 mRNA Vaccinated Immunocompromised Patients.” Frontiers in Immunology 13: 858399. 10.3389/fimmu.2022.858399. 35401575 PMC8988283

[brb370849-bib-0018] Polack, F. P. , S. J. Thomas , N. Kitchin , et al. 2020. “Safety and Efficacy of the BNT162b2 mRNA Covid‐19 Vaccine.” New England Journal of Medicine 383, no. 27: 2603–2615. 10.1056/NEJMoa2034577. 33301246 PMC7745181

[brb370849-bib-0019] Sabatino, J. J., Jr. , A. K. Probstel , and S. S. Zamvil . 2019. “B Cells in Autoimmune and Neurodegenerative central Nervous System Diseases.” Nature Reviews Neuroscience 20, no. 12: 728–745. 10.1038/s41583-019-0233-2. 31712781

[brb370849-bib-0020] Sahin, U. , A. Muik , I. Vogler , et al. 2021. “BNT162b2 Vaccine Induces Neutralizing Antibodies and Poly‐Specific T Cells in Humans.” Nature 595, no. 7868: 572–577. 10.1038/s41586-021-03653-6. 34044428

[brb370849-bib-0021] Schmidt, T. , V. Klemis , D. Schub , et al. 2021a. “Cellular Immunity Predominates Over Humoral Immunity After Homologous and Heterologous mRNA and Vector‐Based COVID‐19 Vaccine Regimens in Solid Organ Transplant Recipients.” American Journal of Transplantation 21, no. 12: 3990–4002. 10.1111/ajt.16818. 34453872 PMC8652989

[brb370849-bib-0022] Schmidt, T. , V. Klemis , D. Schub , et al. 2021b. “Immunogenicity and Reactogenicity of Heterologous ChAdOx1 nCoV‐19/mRNA Vaccination.” Nature Medicine 27, no. 9: 1530–1535. 10.1038/s41591-021-01464-w. PMC8440177 34312554

[brb370849-bib-0023] Schub, D. , M. Fousse , K. Fassbender , et al. 2018. “CTLA‐4‐Expression on VZV‐Specific T Cells in CSF and Blood Is Specifically Increased in Patients With VZV Related Central Nervous System Infections.” European Journal of Immunology 48, no. 1: 151–160. 10.1002/eji.201747079. 28845512

[brb370849-bib-0024] Schub, D. , E. Janssen , S. Leyking , et al. 2015. “Altered Phenotype and Functionality of Varicella Zoster Virus‐Specific Cellular Immunity in Individuals With Active Infection. Research Support, Non‐U.S. Gov't.” The Journal of Infectious Diseases 211, no. 4: 600–612. 10.1093/infdis/jiu500. 25180236

[brb370849-bib-0025] Sormani, M. P. , I. Schiavetti , M. Inglese , et al. 2022. “Breakthrough SARS‐CoV‐2 Infections After COVID‐19 mRNA Vaccination in MS Patients on Disease Modifying Therapies During the Delta and the Omicron Waves in Italy.” EBioMedicine 80: 104042. 10.1016/j.ebiom.2022.104042. 35526306 PMC9069178

[brb370849-bib-0026] Stastna, D. , I. Menkyova , J. Drahota , et al. 2022. “To Be or Not to Be Vaccinated: The Risk of MS or NMOSD Relapse After COVID‐19 Vaccination and Infection.” Multiple Sclerosis and Related Disorders 65: 104014. 10.1016/j.msard.2022.104014. 35803085 PMC9250417

[brb370849-bib-0027] Tarke, A. , C. H. Coelho , Z. Zhang , et al. 2022. “SARS‐CoV‐2 Vaccination Induces Immunological T Cell Memory Able to Cross‐Recognize Variants From Alpha to Omicron.” Cell 185, no. 5: 847–859. 10.1016/j.cell.2022.01.015. 35139340 PMC8784649

[brb370849-bib-0028] Tortorella, C. , A. Aiello , C. Gasperini , et al. 2022. “Humoral‐ and T‐Cell‐Specific Immune Responses to SARS‐CoV‐2 mRNA Vaccination in Patients With MS Using Different Disease‐Modifying Therapies.” Neurology 98, no. 5: e541–e554. 10.1212/WNL.0000000000013108. 34810244 PMC8826460

[brb370849-bib-0029] Wieske, L. , L. Y. L. Kummer , K. P. J. van Dam , et al. 2022. “Risk Factors Associated With Short‐Term Adverse Events After SARS‐CoV‐2 Vaccination in Patients With Immune‐Mediated Inflammatory Diseases.” BMC Medicine 20, no. 1: 100. 10.1186/s12916-022-02310-7. 35236350 PMC8889379

