# Supplementary material for: De Novo Immune Induction After COVID‐19 Vaccination Under B‐Cell Depletion Is Characterized by Robust T‐Cellular Immunity in Patients With Inflammatory Central Nervous System Disease
Source: Brain Behav. 2025 Sep 2;15(9):e70849. doi: 10.1002/brb3.70849 (PMC12405670; doi:10.1002/brb3.70849)
Supplement: Supplementary file 1 — Supplementary Materials: brb370849‐sup‐0001‐SuppMat.docx [file BRB3-15-e70849-s001.docx]

**De novo immune induction after COVID-19 vaccination under B-cell depletion is characterized by robust T-cellular immunity in patients with inflammatory central nervous system disease**

Mathias Fousse^1^, Verena Klemis^2^, Saskia Bronder², Rebecca Urschel², Franziska Hielscher², Klaus Faßbender^1^, Urban Sester^3^, Martina Sester^2^, and Tina Schmidt^2;*^

## ORCID IDs:

Mathias Fousse 0000-0001-5489-1492

Verena Klemis 0000-0002-5636-1273

Saskia Bronder 0000-0001-5863-1268

Rebecca Urschel 0009-0008-2677-5118

Franziska Hielscher 0000-0001-8527-8412

Klaus Faßbender 0000-0003-3596-868X

Urban Sester [0000-0003-4007-5595](https://orcid.org/0000-0003-4007-5595)

Martina Sester [0000-0001-5482-0002](http://orcid.org/0000-0001-5482-0002)

Tina Schmidt 0000-0001-7929-5283

^1^Department of Neurology, Saarland University, 66421 Homburg, Germany; ^2^Department of Transplant and Infection Immunology, Saarland University, 66421 Homburg, Germany; ^3^SHG Kliniken, Völklingen, Germany

*Correspondence: Tina Schmidt, PhD, Saarland University, Department of Transplant and Infection Immunology, Institutes of Infection Medicine, Building 77, Kirrberger Straße, D- 66421 Homburg, Germany; email: [tina.schmidt@uks.eu](mailto:tina.schmidt@uks.eu)

# Supplementary information

**De novo immune induction after COVID-19 vaccination under B-cell depletion is characterized by robust T-cellular immunity in patients with inflammatory central nervous system disease**

Mathias Fousse^1^, Verena Klemis^2^, Saskia Bronder², Rebecca Urschel², Franziska Hielscher², Klaus Faßbender^1^, Urban Sester^3^, Martina Sester^2^, and Tina Schmidt^2;*^

^1^Department of Neurology, Saarland University, 66421 Homburg, Germany; ^2^Department of Transplant and Infection Immunology, Saarland University, 66421 Homburg, Germany; ^3^SHG Kliniken, Völklingen, Germany

*Correspondence: Tina Schmidt, PhD, Saarland University, Department of Transplant and Infection Immunology, Institutes of Infection Medicine, Building 77, Kirrberger Straße, D- 66421 Homburg, Germany; email: [tina.schmidt@uks.eu](mailto:tina.schmidt@uks.eu)

## Figure S1


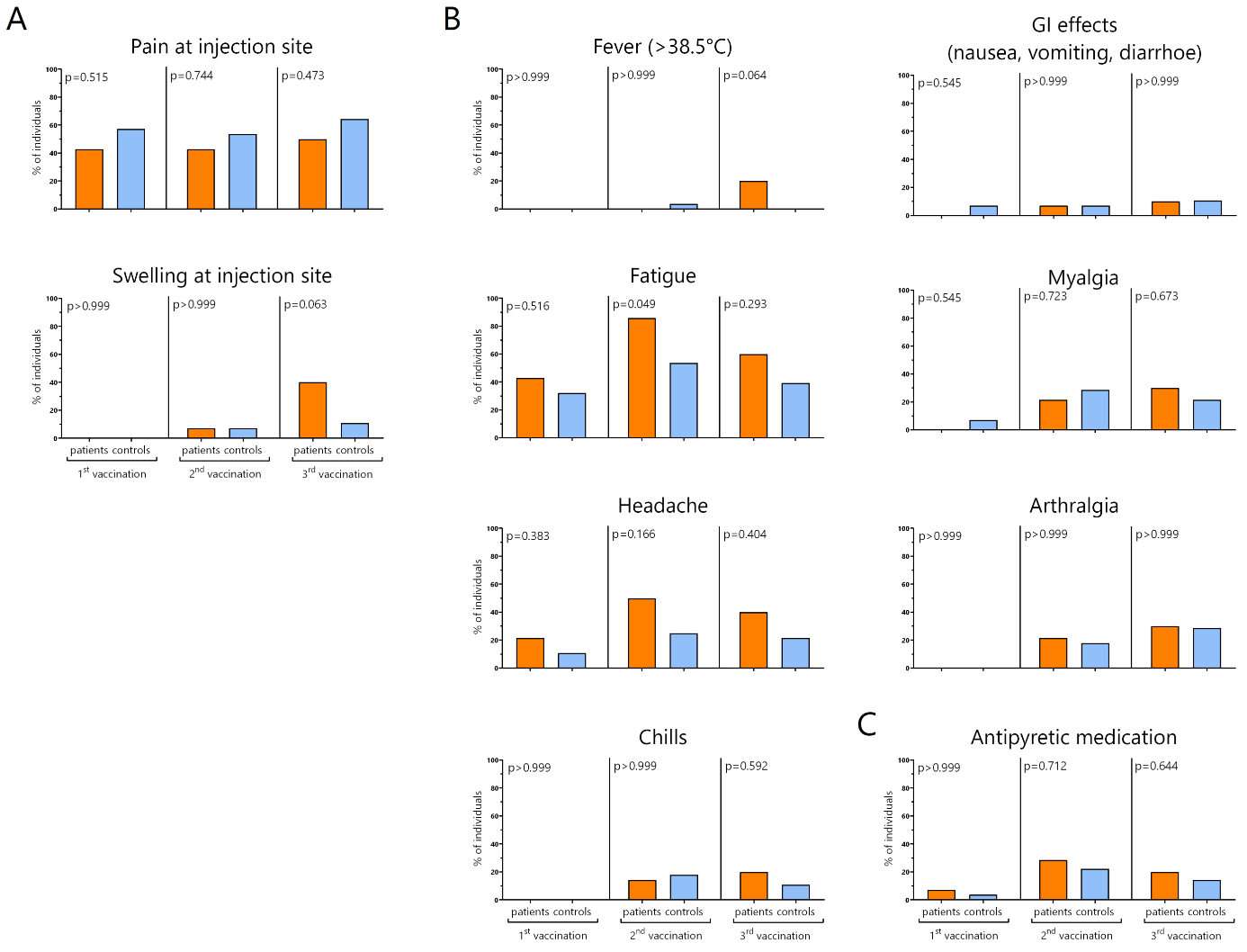


**Figure S1: Detailed comparison of reported reactogenicity after vaccination between B-cell depleted patients and healthy controls.** Results of the self-reporting via standardized questionnaires showing the percentage of individuals reporting the indicated (A) local or (B) systemic events, and (C) the need for antipyretic medication associated with vaccination reactions. Data were available of all 14 patients and 28 controls after first and second vaccination and of 10 patients and 28 controls after third vaccination. The percentages of affected patients (orange) and healthy controls (blue) were compared for each vaccination using a Fisher’s exact test; although fatigue seemed to be slightly more frequent among patients (p=0.049), correction for multiple testing according to Benjamini and Yekutieli with maximum 5% false discovery rate revealed no significant differences between the groups.

## Figure S2


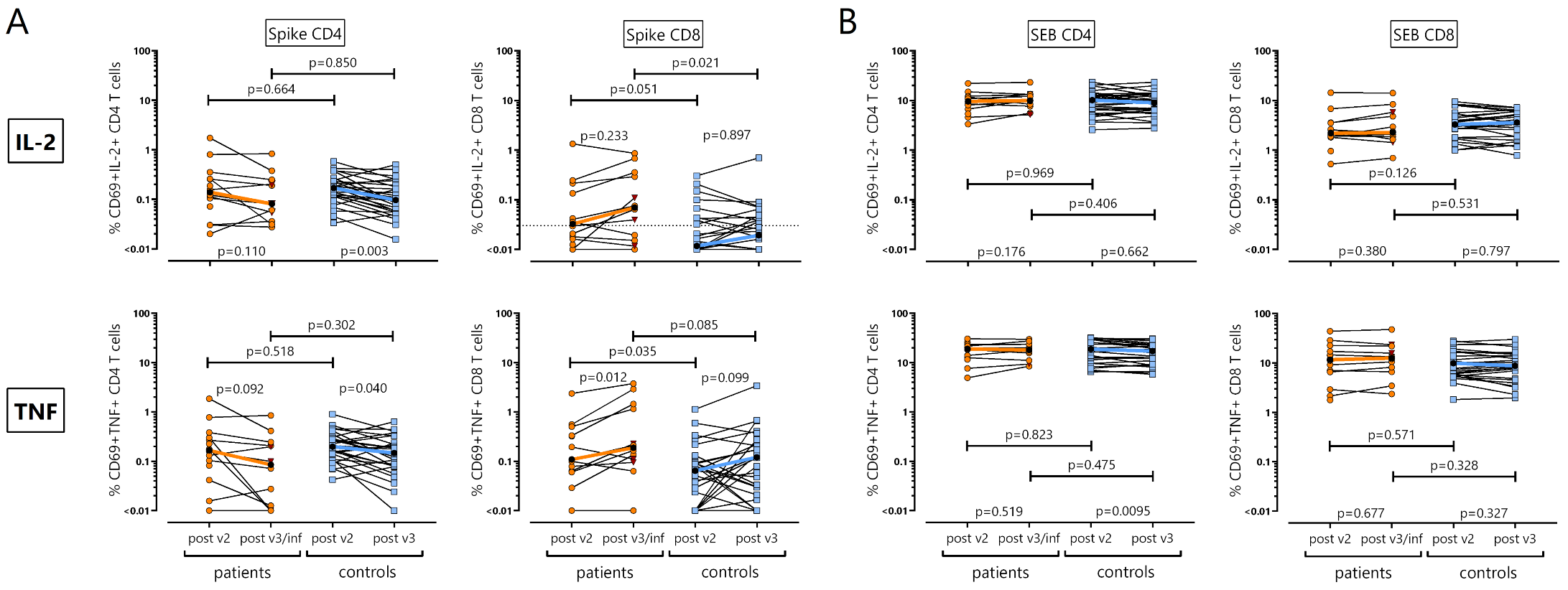


**Figure S2: T-cell reactivity according to expression of IL-2 or TNF.** Percentages of (A) spike-specific and (B) *Staphylococcus aureus* enterotoxin B (SEB)-reactive CD4 and CD8 T cells based on their co-expression of the activation marker CD69 and the cytokine interleukin 2 (IL-2, upper panels) or tumor necrosis factor (TNF, lower panels), respectively, after subtraction of reactivity in the negative control stimulation are depicted after the second vaccination and the third immunization event for B-cell depleted patients (n=14 and n=12 respectively; orange) and controls (n=28; blue) with connecting lines for each individual. (B) Results after infection are represented by red triangular symbols. Bold lines are connecting the respective median values. Statistical analyses of differences between results after second and third vaccination/infection were performed using Wilcoxon signed-rank test including only individuals of which samples for both measurement time points were available. Comparisons between patients and controls at each time point were performed of all available data using Mann-Whitney test. P values <0.05 were considered significant.

## Figure S3


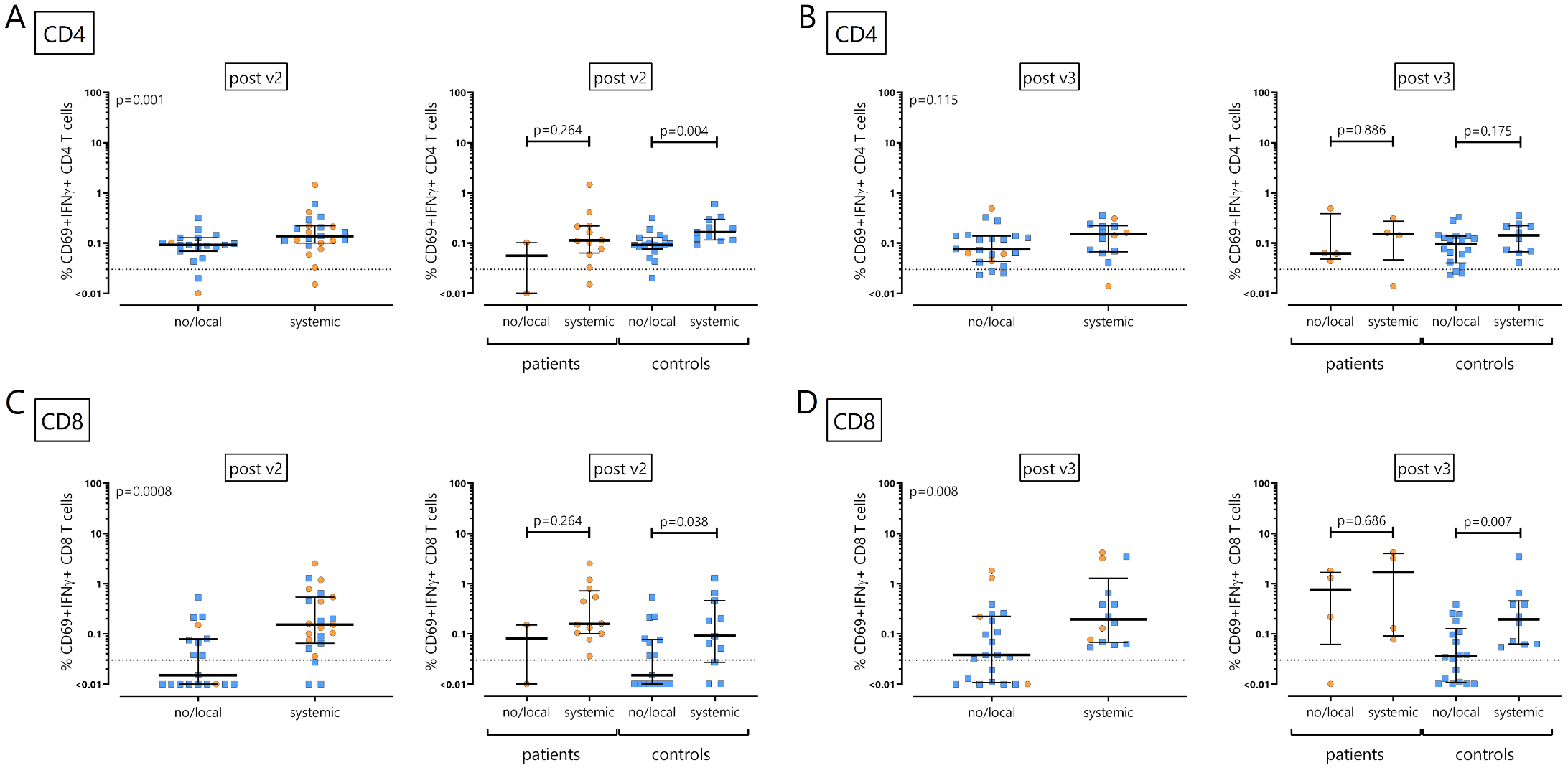


**Figure S3: Association of spike-specific T-cell levels with occurrence of systemic adverse events.** Percentages of spike-specific (CD69+IFNγ+) (A and B) CD4 and (C and D) CD8 T cells after subtraction of reactive cells after negative control stimulation are compared between individuals reporting no or only local adverse advents and individuals reporting systemic adverse events after (A and C) the second and (B and D) the third vaccination. Left panels depict combined analyses for B-cell depleted patients (orange symbols, n=14 after the second and n=8 after the third vaccination) and controls (blue symbols, n=28 after the second and the third vaccination), right panels depict comparisons within each group. Bars represent median and interquartile range. Statistical analyses were performed using Mann-Whitney test. P values <0.05 were considered significant.
